# Transperitoneal laparoscopic retrievement of a migrated prosthetic head after total hip arthroplasty: a case report

**DOI:** 10.3389/fsurg.2023.1227026

**Published:** 2023-07-27

**Authors:** Simone Ciciriello, Martino Gerosa, Riccardo Ghezzi, Angelo Sogni, Ferdinando Incalza, Angelo Guttadauro, Dario Maggioni, Giulio Mari

**Affiliations:** ^1^ General Surgery Residency, University of Milan, Milan, Italy; ^2^ Laparoscopic and Oncological General Surgery Department, Desio Hospital, ASST Brianza, Desio, Italy; ^3^ Orthopaedic Department, Desio Hospital, ASST Brianza, Desio, Italy; ^4^ Department of Anesthesia and Intensive Care, Desio Hospital, ASST Brianza, Desio, Italy; ^5^ Orthopaedic Residency, University of Milano Bicocca, Milan, Italy; ^6^ Department of Medicine and Surgery, School of Medicine and Surgery, University of Milano Bicocca, Milan, Italy

**Keywords:** prosthetic head migration, total hip arthroplasty, laparoscopy, posterolateral approach, abdominal cavity

## Abstract

The migration of a prosthetic head during total hip arthroplasty (THA) is a rare complication. Few cases are described in the literature, offering different solutions and surgical approaches for prosthetic head retrievement. Here, we present a case of successful laparoscopic transperitoneal retrieval of a prosthetic head migrated above the right iliac vein after THA with a posterolateral approach.

## Introduction

The migration of a prosthetic head during total hip arthroplasty (THA) is well described in the literature (1). Head migration can be worsened by the immediate manual attempts to retrieve it (2). The head can thus dislocate within the soft tissues surrounding the prosthesis, through the psoas muscle, or even perforate the pelvic peritoneum and enter the abdominal cavity (3). The treatment of this complication requires the retrieval of the prosthetic head (4). Several case reports have highlighted how this procedure can be performed through an anterior approach to the iliac wing (5–7). However, for pelvic migration with the suspected involvement of the peritoneum, an injury of intra-abdominal organs leading to intraperitoneal contamination could happen (8). A careful intra-abdominal check should be therefore taken into consideration. Here, we present a case of laparoscopic retrieval of a prosthetic head migrated above the right iliac vein after THA.

## Case presentation

**Figure 1:**
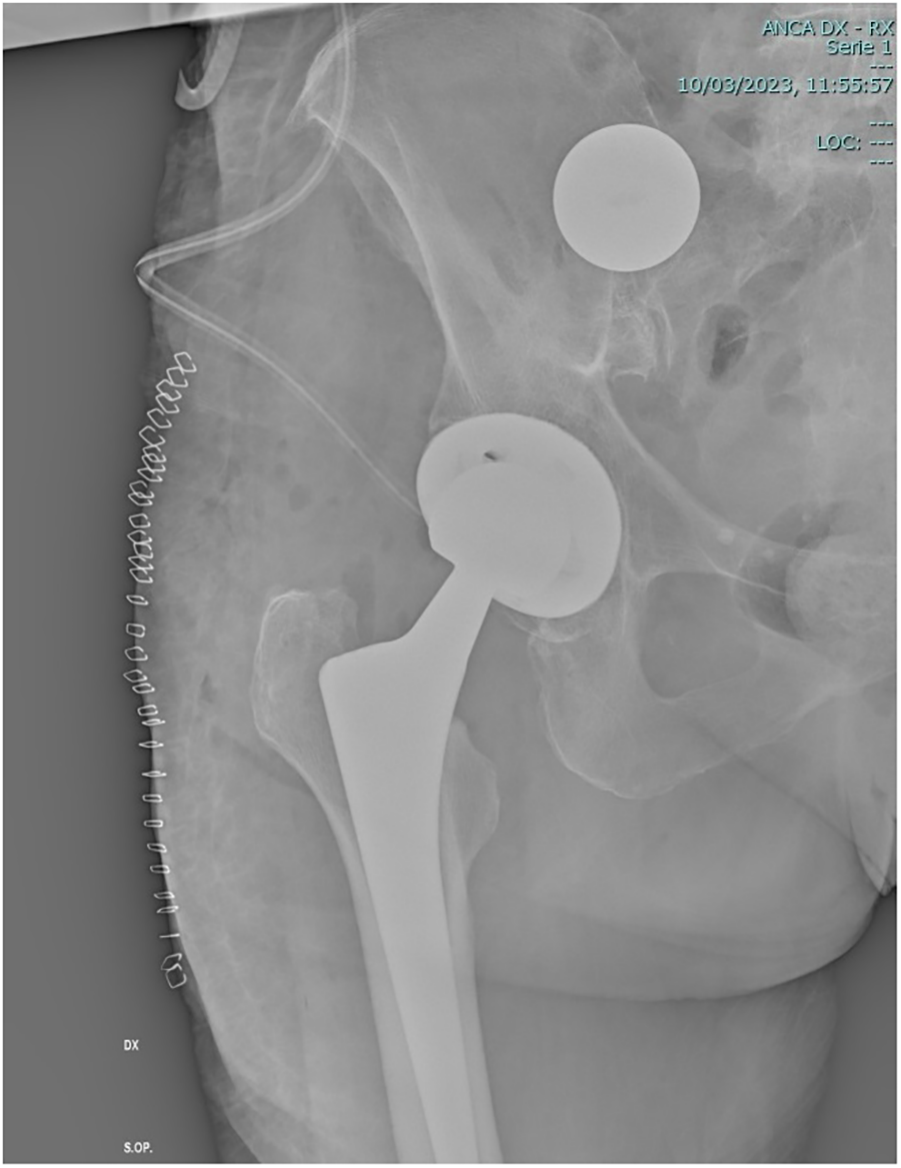
Intraoperative x-ray of the right limb.

A 66-year-old woman patient underwent total hip arthroplasty (THA) using a posterolateral approach following a traumatic femoral neck fracture; she was discharged. After the 10th postoperative day, the patient experienced intense pain (VAS score 8) along the surgical site. During follow-up visits, THA dislocation was not taken into account in the beginning, considering clinical presentation: the limb was not in the typical attitude (flexed and abducted) as it should be expected in common THA dislocation via the posterolateral approach. As the pain symptoms were not regressing, an x-ray of the limb was performed, showing an anterior hip dislocation, along with disengagement of the prosthetic head. Revisional surgery was then proposed: after the first inspection, integrity and cohesion of the femur together with his prosthetic stem were assessed, and the prosthetic head appeared to be above the iliopubic branch, wedged into the iliopsoas muscle (migration through lacuna muscolorum). Intraoperative x-ray indicated the intra-abdominal localization of the ceramic head (Figure 1). All the attempts to manually retrieve the prosthetic head were unsuccessful. The plastic liner with the cup was removed and replaced because the elevated rim line seemed to have triggered the femuroacetabular impingement. Afterward, a new prosthetic head was successfully implanted, and the patient was referred to the general surgeon's attention.

**Figure 2:**
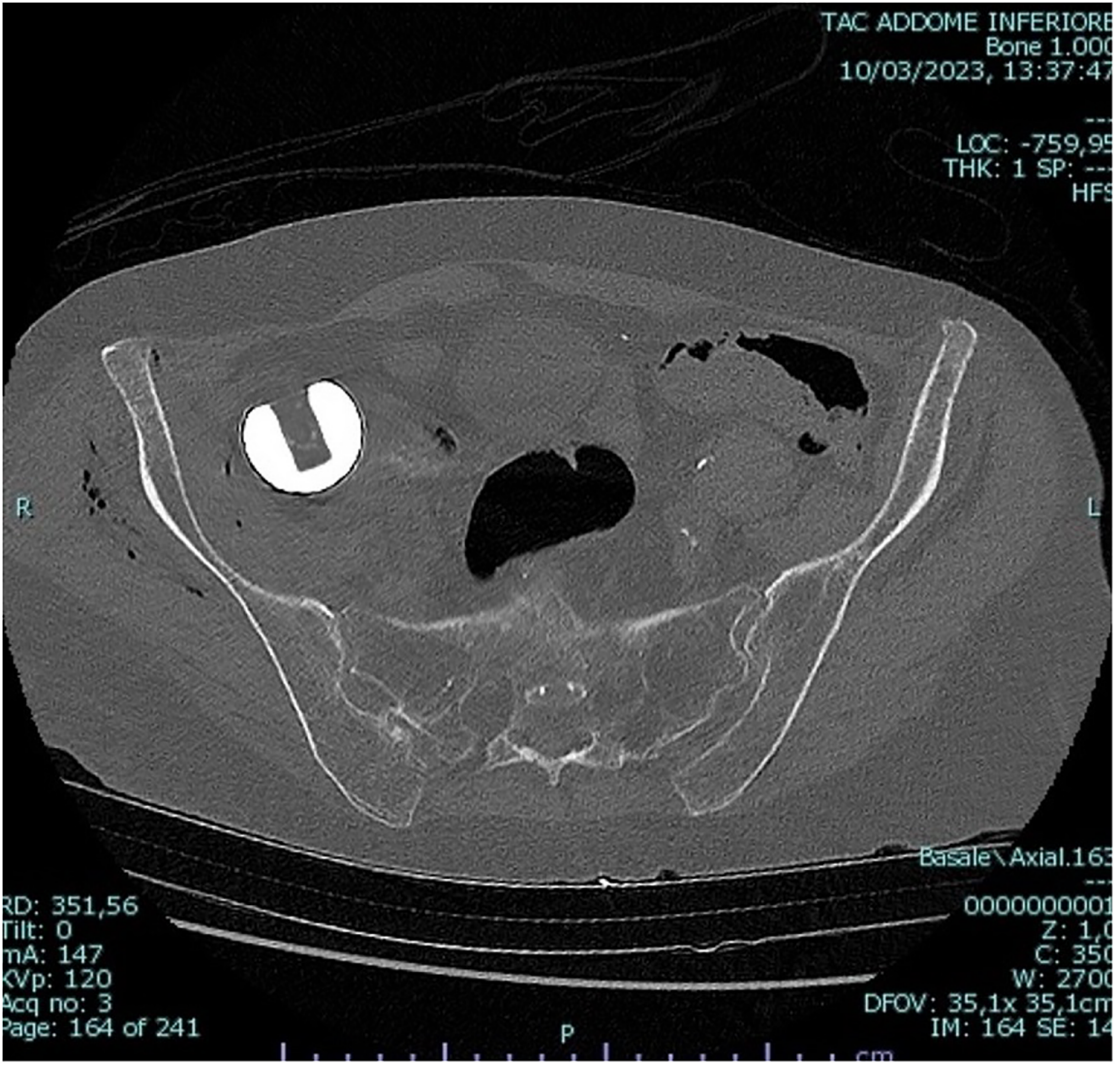
Axial view of the pelvic CT scan.

**Figure 3:**
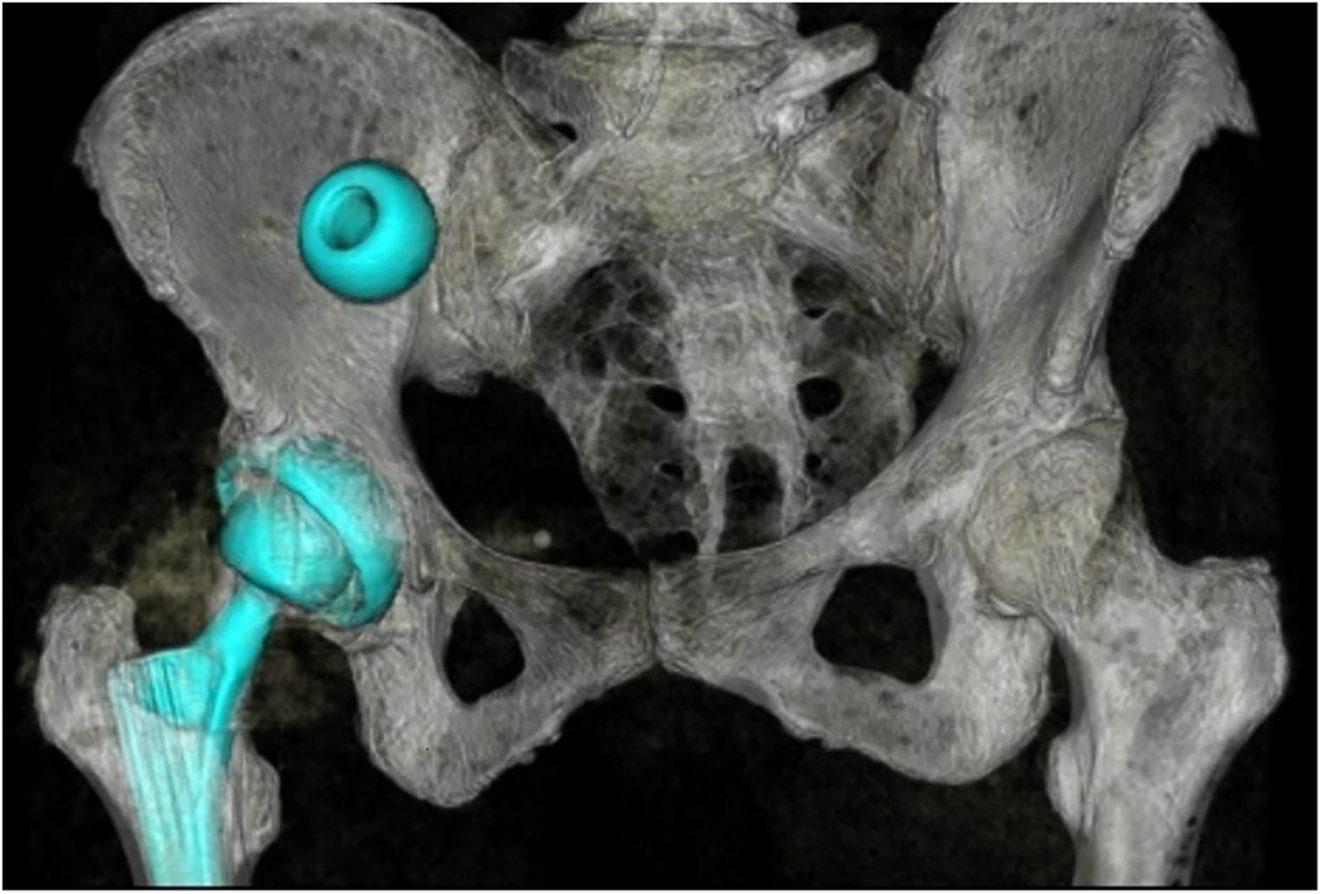
3D reconstruction of the pelvic CT scan.

A pelvic CT scan was performed to highlight the position of the prosthetic head with respect to the pelvic peritoneum and the intra-abdominal organs. The prosthetic head was in direct contact with the right pelvic peritoneum, just above the right iliac vein. No intra-abdominal free air or free fluid was detected (Figures 2, 3). Indication to perform an exploratory laparoscopy was given, and it was executed 4 days after the prosthetic revisional surgery.

A 12-mmHg pneumoperitoneum was obtained via a Verres needle placed in the Palmer point, then three trocars were placed: a 12-mm optic trocar in the left iliac fossa, a 5-mm trocar in the supraumbilical location, and a 10-mm trocar in the hypogastrium. After having listed multiple omentoparietal adhesions (as a result of a previous cesarean section), under the peritoneal veil, a bulge of the peritoneum in the right iliac fossa, close to the iliac vein, was detectable. As the finding was consistent with the radiological report, a decision was made to mobilize the cecum and begin with the dissection of the peritoneum overlying the bulge for a 3 cm length. The ceramic head was then detected and collected in a laparoscopic endobag. No signs of extraperitoneal inflammation were present, tissues were adequately irrigated, and the peritoneal opening was closed using an intracorporeal running suture. The endobag was then removed through the hypogastric port (Supplementary Video S1). No surgical drain was left in the abdominal cavity. The postoperative course was uneventful, and the patient was discharged 3 days after surgery to begin a rehabilitation program.

## Discussion

Complications during THA surgery are mostly expected during stability testing. Cases of anterior dislocation and migration of the femoral head days after THA via posterolateral access are sporadic. A literature review showed cases involving trial heads and just one reported case of femoral head dislodgement after closed THA dislocation (4). In similar cases, when the prosthetic head lays on the anterior rim of the acetabulum, close attention is needed to avoid a pelvic migration while trying to retrieve it, and the possibility of anterior dislocation post-THA should be considered.

Intrapelvic migration of the femoral head should not be related to the surgical approach since such a complication is related to posterior, lateral, and anterior incisions. Pita et al., in fact, hypothesized that femoral head displacement could happen within the reduction maneuvers, while assessing the anterior stability, or during dislocation after completing the implant trialing (6).

The anatomical plane right above the psoas muscle could be the migration path of the prosthetic head toward the retroperitoneal space; until now, there has been only one reported case of intraperitoneal migration (8). In most cases, the intraoperative attempts to grasp the lost implant may inadvertently push it further into the pelvis (9). Most of the attempts of head removal reported in the literature involved an open approach that allowed access to the extraperitoneal space of the pelvis (3, 4, 10, 11).

Only one case report refers to a laparoscopic removal of a migrated prosthetic head. Alfonso et al., in fact, depicted a laparoscopically diagnosed hemoperitoneum and a small abdominal wall defect in the right lower quadrant without bowel injury (8).

The use of the laparoscopic technique to explore the peritoneal cavity is a well-established practice (12). The pelvic exposure can be easily obtained through a standard periumbilical port. The anatomical structures that can be injured due to a foreign body migration from the coxofemoral region to the pelvic entrance are the iliac vein and artery, the hypogastric vein, and the ureter. All these structures are easily displayable by an exploratory laparoscopy, while an anterior laparotomic approach could not be readily identified them. It stands to reason that if a CT scan describes the migrated prosthetic head nearby the iliac vessels, laparoscopy could easily detect it. Therefore reaching an extraperitoneal foreign body may be easier via the transperitoneal approach. Moreover, laparoscopy allows to secure the nearby organs and visualize the extraperitoneal structures through the peritoneum. The opening of the peritoneum itself, as well as its closure, is also performed under direct vision. Given the possibility of intra-abdominal contamination, it is by these means possible to perform a peritoneal lavage and possibly place a drain.

## Conclusion

The transperitoneal laparoscopic approach is safe and effective for retrieval of a migrated prosthetic head after THA. Intra-abdominal checks should be taken into consideration for migrations involving the pelvic peritoneum.

## Data Availability

The raw data supporting the conclusions of this article will be made available by the authors without undue reservation.
